# Adhesion and Anti-Adhesion Abilities of Potentially Probiotic Lactic Acid Bacteria and Biofilm Eradication of Honeybee (*Apis mellifera* L.) Pathogens

**DOI:** 10.3390/molecules27248945

**Published:** 2022-12-15

**Authors:** Aleksandra Leska, Adriana Nowak, Karolina Henryka Czarnecka-Chrebelska

**Affiliations:** 1 Department of Environmental Biotechnology, Lodz University of Technology, Wolczanska 171/173, 90-530 Lodz, Poland; 2 Department of Biomedicine and Genetics, Medical University of Lodz, 5 Mazowiecka Str. (A-6 Building), 92-215 Lodz, Poland

**Keywords:** lactic acid bacteria, adhesion, anti-adhesion, honeybee pathogens, *Paenibacillus larvae*, *Melissococcus plutonius*, biofilm eradication

## Abstract

Lactic acid bacteria (LAB) naturally inhabits the organisms of honeybees and can exhibit adhesive properties that protect these insects against various pathogenic microorganisms. Thus, cell surface (auto-aggregation, co-aggregation, hydrophobicity) and adhesive properties of LAB to two abiotic (polystyrene and glass) and four biotic (collagen, gelatin, mucus, and intestinal Caco-2 cells) surfaces were investigated. Additionally, anti-adhesion activity and the eradication of honeybee pathogen biofilms by LAB metabolites (culture supernatants) were determined. The highest hydrophobicity was demonstrated by *Pediococcus pentosaceus* 19/1 (63.16%) and auto-aggregation by *Lactiplantibacillus plantarum* 18/1 (71.91%). All LAB showed a broad spectrum of adhesion to the tested surfaces. The strongest adhesion was noted for glass. The ability to co-aggregate with pathogens was tested for the three most potently adherent LAB strains. All showed various levels of co-aggregation depending on the pathogen. The eradication of mature pathogen biofilms by LAB metabolites appeared to be weaker than their anti-adhesive properties against pathogens. The most potent anti-adhesion activity was observed for *L. plantarum* 18/1 (98.80%) against *Paenibacillus apiarius* DSM 5582, while the strongest biofilm eradication was demonstrated by the same LAB strain against *Melissococcus plutonius* DSM 29964 (19.87%). The adhesive and anti-adhesive activity demonstrated by LAB can contribute to increasing the viability of honeybee colonies and improving the conditions in apiaries.

## 1. Introduction

Lactic acid bacteria (LAB) have been widely used as probiotics due to their beneficial effects on the health of the host, such as improved digestion, strengthening the immune system, and the mucosal barrier [1]. An expert panel organized by the International Scientific Association for Probiotics and Prebiotics (ISAPP) in October 2013 defined probiotics as “live microorganisms, which, when administered in adequate amounts, confer a health benefit on the host” [2]. Probiotic LAB modulates various biological functions and, through antagonistic activity, can inhibit the growth of pathogens that cause diseases in the host’s body [3]. The adhesion capacity of LAB plays a key role in preventing the invasion and colonization of gut pathogens and determines the competitive abilities of these bacteria [4]. Adhesion is the interaction between the complementary structure of the host cell surface and the surface components of the bacterial cell wall [5]. A potential mechanism related to the LAB adhesion ability is the production of exopolysaccharides (EPS), lipids, enzymes, carbohydrates, membrane-bound receptors, and nucleic acids [6]. Furthermore, adhesion is recognized as the first step in the production of biofilms. The correlation between the values of adhesion and biofilm formation varies depending on the species of the microorganism. [7]. The attached bacteria are able to metabolize the substrates bound to the surface and then grow in size and reproduce [8]. Additionally, biofilm-forming microorganisms demonstrate increased resistance to antimicrobial agents [7]. Factors influencing bacterial adhesion include the physical conditions of the medium (e.g., the presence of proteins, carbohydrates, bactericidal substances, or serum proteins) and the physical nature of the material [9]. Biofilms that are produced by microorganisms can develop on many surfaces, including metal, plastic, wood, soil particles, stainless steel, and biotic materials. LAB adheres to and forms biofilms on abiotic and biotic surfaces to function as antagonistic effectors [10]. The antagonistic activity of bacteria is also related to their metabolism. LAB produces metabolites such as organic acids, vitamins, amino acids, mannitol, EPS, and bacteriocins. EPS from LAB exhibits anti-biofilm, anti-viral, anti-bacterial, and anti-inflammatory activity [11]. The anti-biofilm activity of EPS inhibits the formation of the biofilms of pathogens such as *Escherichia coli*, *Staphylococcus aureus,* and *Salmonella enterica* subsp. *enterica* serovar Typhimurium [12]. LAB also produces 2-hydroxyisocaproic acid, which inhibits the biofilm formation of certain microorganisms’ planktonic cells, showing the most potent effects at an acidic pH [13]. The production of antimicrobial compounds by LAB has the potential to inhibit the growth of bacterial biofilms of pathogens [14].

Honeybees (*Apis mellifera* L.) are important pollinators with a significant impact on global food supplies and the agricultural economy [15]. The microbiota plays an important role in the health of honeybees by performing various antimicrobial functions and promoting host weight gain via hormonal signaling and bacterial metabolism [16]. Under unstressed conditions, the honeybee microbiome contributes to the erection of the barrier against microbial diseases [17]. Comparative analysis suggests that different species of bacteria (including LAB) inhabiting the honeybee’s gut exhibit distinct functional capabilities related to carbohydrate breakdown, host interaction, and biofilm formation [18]. LAB are able to colonize the intestine of honeybees and form networks and biofilms, contributing to the protection of these pollinators from various health risks [6]. LAB, when isolated from the organism of the honeybee, demonstrates various intensities of biofilm production depending on environmental conditions. Strains of the species *Apilactobacillus kunkeei,* which also show antagonistic activity against honeybee pathogens, exhibit a particularly potent ability to form biofilms in the presence of fructose [19]. The genome of *A. kunkeei* encodes proteins that act similar to antibacterial compounds and are possibly involved in the production of biofilms [20]. LAB strains can produce various biosurfactants that modify the integrity of the cell envelope [21]. Changes in the cellular envelope components through interactions with LAB biosurfactants may lead to disturbances in biofilm formation and affect the eradication of mature pathogen biofilms [21]. The ability of LAB to co-aggregate with biofilms of pathogenic bacteria is also one of their important anti-biofilm properties [10]. Honeybees are exposed to bacterial, fungal, viral, and microsporidial pathogens that cause various diseases in the organisms of these pollinators [22]. American foulbrood, caused by the pathogenic bacterium *Paenibacillus larvae*, is one of the diseases that particularly threaten the viability of *A. mellifera* L. This bacterial pathogen infects honeybees in the early stages of development and contributes to brood mortality through the secretion of chitin-degrading enzymes and secondary metabolites that allow the invasion of hemocoel [23]. Genes for building flagella are present in the genome of *P. larvae* and suggest that this microorganism is capable of coordinated activity such as biofilm formation [24]. The exact mechanism of biofilm formation by other honeybee pathogens, such as *Melissococcus plutonius,* has not yet been investigated [25].

Due to the economic and environmental importance of *A. mellifera* L., there is a growing need to find a way to combat pathogens that weaken the viability of these insects. LAB are Generally Recognized as Safe (GRAS) microorganisms, making them good candidates for probiotics with health-promoting functions [26]. The ability of LAB to produce biofilm allows the microorganisms to prevail in the host organism. Thus, our research focuses on determining the adhesion capacity of LAB strains on different origins (mainly isolated from honeybee environments) to various surfaces. According to the authors’ knowledge, there are no in-depth studies on the adhesive properties of LAB isolated from the honeybee environment. Thus, the aim of this study was to determine their adhesion to abiotic (polystyrene and glass) and biotic (collagen, gelatin, mucus, and Caco-2 cells) surfaces. Additionally, the influence of LAB and their metabolites on the biofilms of honeybee pathogens has not been thoroughly investigated. In our study, we examined the effect of LAB culture metabolites on the first step of biofilm production (i.e., adhesion) and the eradication of mature biofilms of honeybee pathogens. Potentially probiotic LAB demonstrating a high adhesion capacity may contribute to strengthening the resistance of these pollinators. The anti-adhesive activity of LAB offers interesting prospects in the prevention of infections that increase colony mortality. The results of our study may contribute to the construction of a probiotic preparation increasing the survival rate of honeybees exposed to various diseases.

## 2. Results and Discussion

### 2.1. Cell Surface Properties of LAB Strains: Auto-aggregation, Coaggregation and Hydrophobicity

In the present study, we determined the auto-aggregation ability of 20 LAB strains (Table 1). *A. kunkeei* DSM12361 isolated from the digestive tract of honeybees demonstrated a lower auto-aggregation capacity compared to the rest of the tested LAB strains (except for *Pediococcus acidilactici* 4/1 and *Lactiplantibacillus plantarum* 145). The auto-aggregation of bacteria is related to their persistence and survival in the host’s body. The ability of LAB to aggregate corresponds to their ability to adhere to cells, the production of biofilms, and the displacement of pathogens [27,28]. In our study, the auto-aggregation demonstrated by LAB ranged from 27.74% ± 4.50% for *P. acidilactici* 4/1 to 71.91% ± 5.44% for *L. plantarum* 18/1. According to Tuo et al., *Lactobacillus rhamnosus* GG showed a higher capacity of auto-aggregation than the remaining 21 LAB strains tested [29]. The results of the current study showed that the auto-aggregation of *L. plantarum* 10/2 (61.61% ± 3.12%) and 21/1 (66.63% ± 3.00%), *P. acidilactici* 18/1 (71.91% ± 5.44%), 25/1 (65.04% ± 0.90%), *Pediococcus pentosaceus* 19/1 (68.04% ± 5.31%), and *Pediococcus parvulus* OK-S (65.56% ± 2.71%) was higher than the auto-aggregation capacity of *L. rhamnosus* GG (60.83% ± 0.75%), which was used as a positive control. This suggests the potential of these strains to produce biofilms on intestinal epithelial cells; however, this should be confirmed by in vivo tests. Grigoryan et al. determined the ability of auto-aggregation in the various LAB strains [30]. *L. rhamnosus* INA-5.1, *Lactobacillus helveticus* NRA-2010-H11, and *Lactobacillus acidophilus* JM-2012 showed a high auto-aggregation from approximately 46% to 73%, with results similar to our study [30]. In vitro tests carried out by Zawistowska-Rojek et al. demonstrated that the highest auto-aggregation (21.4%) was shown by *L. acidophilus* LaK when isolated from a probiotic product [31]. *L. rhamnosus* LrB and LrC showed a similar auto-aggregation capacity for *L. rhamnosus* GG when used as the control strain [31]. The aggregation of some LAB strains depends on environmental conditions. Saito et al. suggested that aggregation in the presence of glucose is dependent on the LAB strain tested [32]. Contrary to *Levilactobacillus brevis* NBRC 13109, 13110, and 3960, which showed a high auto-aggregation capacity in glucose, no aggregation was observed for *L. brevis* NBRC 120005, 107147T and 12005 [32]. The ability to aggregate in the presence of carbohydrates is an important feature for potential probiotic LAB to enhance the viability of honeybees due to the supplementation of sugar syrups as food for these insects [33].

Past studies have suggested that the auto-aggregation capacity of bacteria does not correlate with their levels of surface hydrophobicity [29,34]. This conclusion can also be drawn from our study, where no correlation was observed between the auto-aggregation and hydrophobicity of the tested LAB strains, except for *L. plantarum* species (r = 0.74, Spearman’s rank correlation coefficient). The adhesive and coaggregation properties of LAB can be estimated by their hydrophobicity which is evaluated by the affinity of bacteria to solvents such as toluene, xylene, or hexane [35,36]. The relative hydrophobicity of LAB depends on the method used [37]. According to Arellano-Ayala, the auto-aggregation and hydrophobicity of LAB strains correlated with their bacterial adhesion to the tomato surface [35]. Probiotic bacteria should exhibit basic properties such as auto-aggregation and hydrophobicity [38]. In the present study, *P. acidilactici* 4/1, *Lacticaseibacillus casei* 12AN, *L. plantarum* 145, and *L. rhamnosus* ŁOCK 0908 demonstrated lower hydrophobicity than the probiotic *L. rhamnosus* GG (Table 1). The highest hydrophobicity was demonstrated by *L. plantarum* 18/1 (52.45% ± 2.80%), *P. pentosaceus* 19/1 (63.16% ± 5.27%), and *P. acidilactici* 25/1 (73.49% ± 2.72%), suggesting the potentially probiotic properties of these strains. The probiotic properties of *P. pentosaceus* 19/1 and P. *acidilactici* 25/1 were significantly elevated compared to *P. acidilactici* 4/1 (*p* = 0.044; *p* = 0.015, respectively; Kruskal–Wallis test). *A kunkeei* DSM 12361 demonstrated lower hydrophobicity (18.87% ± 0.94%) compared to most of the LAB strains tested. During in vitro tests, Somashekaraiah et al. investigated the cell surface hydrophobicity of 7 LAB strains using xylene [38]. LAB strains demonstrated different levels of hydrophobicity: from 51.10% for *Enterococcus lactis* MYSN 43 to 77.82% for *L. brevis* MYSN 106 [38]. Guan et al. determined the hydrophobicity of six LAB strains isolated from the human intestinal tract of the longevous population from China [39]. The hydrophobicity spectrum ranged from 14.8% for *Streptococcus thermophilus* 90–57 to 57.3% for *L. casei* g9 [39].

### 2.2. Adhesive Properties of LAB

**Figure 1:**
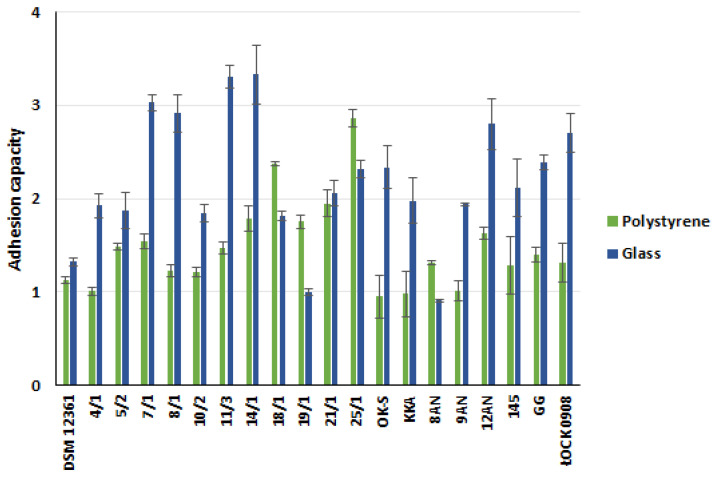
The capacity of lactic acid bacteria (LAB) to adhere to abiotic surfaces. Adhesion capacity: A ≤ 1 no adhesion; 2 ≥ A > 1 weak adhesion; 3 ≥ A > 2 medium adhesion; A > 3 strong adhesions. Each data point represents the means from four individual wells. Results are presented as mean ± standard deviation (S.D.).

**Figure 2:**
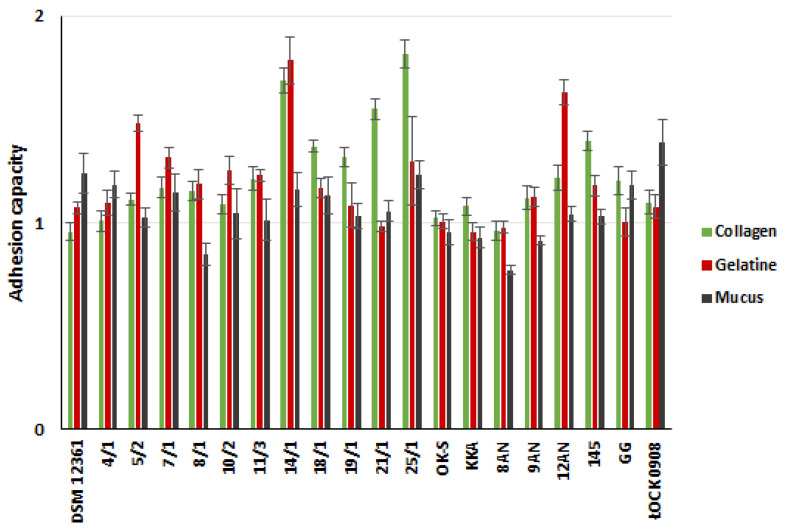
The capacity of lactic acid bacteria (LAB) to adhere to biotic surfaces. Adhesion capacity: A ≤ 1 no adhesion; 2 ≥ A > 1 weak adhesion; 3 ≥ A > 2 medium adhesion; A > 3 strong adhesions. Each data point represents the means from four individual wells. Results are presented as mean ± standard deviation (S.D.).

In this experiment, we assessed the adhesion capacity of 20 LAB strains to two abiotic (glass and polystyrene) and three biotic (mucus, gelatin, and collagen) surfaces (Figure 1 and Figure 2, Table S1 in the Supplementary Materials). *A. kunkeei* DSM 12361 strain, which naturally inhabits the honeybee gut, was used as a control strain. LAB adhered to the tested surfaces at different degrees, and the bacteria’s origin did not affect the adhesion ability. All tested bacterial strains adhered more potently to abiotic surfaces, with the highest values of adhesion capacity observed for glass. *A. kunkeei* DSM 12361 demonstrated weak or absent adhesion ability to all the surfaces tested. Fructophilic bacteria of the species *A. kunkeei* form abundant biofilms in the digestive tract of honeybees and may also facilitate metabolic processes, reduce pathogen loads, and improve the barrier function of insect microbiota [40]. The cellular adhesion of *A. kunkeei* depends on the environment, surface area, contact time, and the sugar content of the medium [41,42]. According to Iorizzo et al., *A. kunkeei* DSM 12361 displayed a higher adhesion capacity in MRS medium without sugar compared to media supplemented with glucose, fructose, and sucrose [42]. Exposing honeybees to certain chemicals may change the composition of LAB biofilms and cause a reduction in cell density [40]. The sensitivity of the biofilms produced by LAB inhabiting the digestive tract of honeybees suggests the need to strengthen the microbiota of these insects with additional, more resistant probiotic microorganisms. In the above study, the adhesion ability of LAB was a strain-dependent feature. Most of the LAB strains tested showed weak adhesion to abiotic surfaces; however, the glass adhesion values were the highest among all the surfaces tested. In our study, glass and polystyrene were used due to their frequent use as model surfaces to test the adhesion capacity of bacteria [43,44,45]. *P. pentosaceus* 11/3 showed a significantly potent glass adhesion capacity (*p* < 0.05, Kruskal–Wallis test). Bacteria promote the adhesion of a substrate that is similar to their own surface charge [46]. In a study conducted by Wallis et al., *L. brevis* 104/37 showed the strongest biofilm production on glass [47]. The optical density values of the samples containing Tween 80 (hydrophilic surfactant) were dependent on the LAB strain, suggesting a relationship between biofilm formation and the charge of the examined surface [47]. Moreover, LAB biosurfactants reduced the adhesion of pathogenic bacteria to the glass [48]. Competition between microorganisms can reduce the risk of diseases caused by pathogens in honeybees [49].

The current study also determined the adhesion capacity of LAB strains to polystyrene, which, similar to glass, has a hydrophobic surface. *L. plantarum* 18/1 and *P. acidilactici* 25/1 showed significantly stronger adhesion capacities to the polystyrene in comparison to other LAB strains (*p* < 0.05, KW test) (Table S1 in Supplementary Materials). In a study by Sepova et al., all LAB strains demonstrated the ability to adhere to polystyrene regardless of the charge of the bacterial surface [50]. Similar conclusions were presented by Balcazar et al. [51]. *Latilactobacillus curvatus* CLFP 150, *Lactococcus lactis* subsp. *lactis* CLFP 100, *Latilactobacillus sakei* CLFP 202, *L. lactis* 73 subsp. *cremoris* CLFP and *Leuconostoc mesenteroides* CLFP 196 showed a strong adhesion capacity to polystyrene [51]. Their adhesion to polystyrene may depend on environmental conditions. According to Haddaji et al., the polystyrene adhesion capacity of LAB decreases at a low pH [52]. The ability of the LAB to adhere in an acidic environment is an important feature for performing probiotic functions in the host gastrointestinal tract. The surface properties of collagen and gelatin depend on charged groups containing hydrophilic and hydrophobic amino acids [53]. The adhesion capacity of these surfaces depends on the amino acid composition, which can affect the barrier and mechanical properties of the biofilms [54]. In medical and pharmaceutical applications, probiotic LAB strains are often contained in gelatin-based coatings, and it is advisable that the bacteria show a high adhesion ability to this surface [55]. The capacity of potentially probiotic bacteria to adhere to collagen is significant due to its presence in the gastrointestinal tract and expression in intestinal epithelial cells [56]. Similarly, in our study, the most potent adhesion to gelatin was noted by *P. pentosaceus* 14/1, which also demonstrated a significant collagen adhesion capacity compared to the control strains (*p* <0.05, KW test) (Table S1 in the Supplementary Materials). A slightly higher adhesion to collagen was exhibited by *P. acidilactici* 25/1. Collagen-binding proteins are present in some LAB strains and condition adhesion to this surface [57]. According to the adhesion assay carried out by Yadav et al., *L. plantarum* 91 showed a strong collagen adhesion capacity, and purified collagen-binding proteins (Cbp) were found to significantly affect the adhesion of bacteria [58]. Moreover, this protein was also responsible for the anti-adhesive activity against enteropathogenic *E. coli* 0157: H7 on collagens, reducing the ability to form biofilms by 59.71% compared to the control well [58]. Gelatin is a soluble, degraded form of collagen that is obtained by partial hydrolysis [59]. The comparable results achieved in the present study for these surfaces may be due to their similarities. Some LAB exhibit gelatinase activity without passing safety tests as potential probiotics [60]. In vitro tests by Gomez et al. showed no degradation of gelatin in all the tested LAB strains [48]. This suggests that the adhesion capacity of gelatin is determined by the LAB enzymatic activity and is individual for the strain, as demonstrated by the results of the above study. The significantly potent ability to adhere to intestinal mucus was demonstrated by *L. rhamnosus* ŁOCK 0908 (*p* < 0.05, KW test) (Table S1 in the Supplementary Materials), which in previous studies showed poor adhesion to the tested surfaces [61,62]. It may be due to the fact that this strain was isolated from human feces. *L. rhamnosus* ŁOCK 0908 also produces an extracellular slime-like substance, which may facilitate its adhesion to mucus and hinder it from adhering to other surfaces [63]. Due to the low adhesion ability of this strain, we selected it as a negative control in our study. Intestinal epithelial cells are covered with a layer of mucus, which plays a crucial role in protecting the digestive tract of mammals. The ability to adhere to mucus is a significant probiotic function of LAB [64]. Sugimura et al. suggested a correlation between LAB’s colonization ability in vivo and in vitro adhesion to carp intestinal mucus [65]. LAB strains showing a stronger adhesion to the slippery surface remained in the alimentary path for several weeks, and strains demonstrating poor adhesion disappeared shortly after the supplementation of food was stopped [65]. Contrary to the results of the above experiment, the adhesion assay conducted by Li et al. demonstrated the high intestinal mucus adhesion ability of four tested LAB strains (*L. gasseri* S1031, *Limosilactobacillus reuteri* I202, *L. acidophilus* I021 *and Limosilactobacillus fermentum* I5007) [66]. In our study, all LAB strains showed a relatively similar or greater adhesion capacity than *L. rhamnosus* GG, which in previous studies, demonstrated a potent adhesion to various surfaces [66,67].

**Figure 3:**
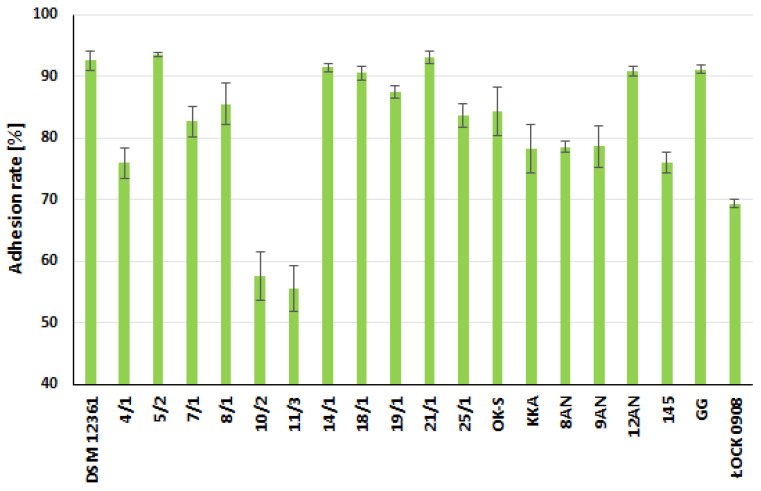
Adhesion of lactic acid bacteria (LAB) to Caco-2 cell monolayer after 2 h incubation. The presented results are the mean from two repeats ± standard deviation (SD).

**Figure 4:**
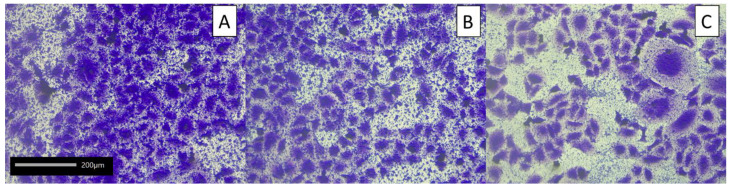
Selected microphotographs showing adhesion of lactic acid bacteria to Caco-2 cells: (**A**) *L. plantarum* 18/1, (**B**) *L. plantarum* 21/1, and (**C**) *P. acidilactici* 25/1 (objective 20×). Observed under a phase-contrast microscope (Nikon Eclipse Ci H600L, Tokyo, Japan) attached to a digital camera (NikonDigital Sight DS-U3, Tokyo, Japan) and imaging software (NIS-elements BR 3.0, Nikon, Tokyo, Japan).

Potentially probiotic LAB strains have also been tested for their ability to adhere to model intestinal epithelial Caco-2 cells (Figure 3 and Figure 4, Table S1 in the Supplementary Materials). This cell line is widely used as an in vitro research intestinal barrier model [68]. The strains showing the strongest adhesion capacity were *A. kunkeei* DSM 12361, *P. acidilactici* 5/2, *P. pentosaceus* 14/1, and *L. plantarum* 21/1, where the adhesion rate was 92.50% ± 1.55%, 93.48% ± 0.37%, 91.39% ± 0.61%, and 93.03% ± 0.94%, respectively. These bacteria also showed a stronger adhesion ability compared to the probiotic *L. rhamnosus* GG strain. *P. acidilactici* 5/2 and *L. plantarum* 21/1 exhibited significantly higher adhesive properties in comparison to *L. plantarum* 10/2 and *P. pentosaceus* 11/3 (*p* < 0.05, KW test) (Table S1 in the Supplementary Materials).

In general, the adhesion rate values ranged from 55.50% ± 3.37% for *P. pentosaceus* 11/3 to 93.48% ± 0.37% for *P. acidilactici* 5/2. Following the in vitro tests conducted by Wang et al., five LAB strains (*L. plantarum* BSGP201683 (G83); *Weissella confuse* WJ202009 (W9), BSP201703 (X3), WJ202003 (W3), WJ202021 (W21)) derived from the giant panda (*Ailuropoda melanoleuca*) showed a strong ability to form biofilms and adhere to the Caco-2 cell line, suggesting the potentially probiotic properties of these bacteria [69]. According to Hernandez-Alcantara et al., all tested LAB strains adhered more potently to Caco-2 cells compared to the probiotic *L. rhamnosus* GG [70]. LAB strains exhibiting higher adhesion rates than a known probiotic offer interesting prospects for their use as potential support for honeybee microbiota against external threats such as pathogens or pesticides. Morita et al. suggested that despite the ability to form biofilms, LAB did not trigger inflammatory responses in Caco-2 cells [71]. The contact of LAB strains did not stimulate the secretion of the cytokines IL-6 and IL-8. The lack of correlation between cytokine induction and biofilm production does not affect the positive effects of bacterial adhesion [71]. Strains strongly adhering to various surfaces show the potential to be used as probiotics to protect the health and viability of honeybee colonies.

### 2.3. Coaggregation of LAB with Honeybee Pathogens

The recognition and adhesion of certain genera and species of bacteria to one another is known as coaggregation and can be linked to the development of multi-species biofilms [72]. LAB, exhibiting high coaggregation capacity, plays an important role in defending the host organism against infections due to the greater likelihood of forming biofilms to prevent colonization by pathogens [73]. The mechanism of LAB coaggregation influences the elimination of pathogens from the host body. A high level of coaggregation may also facilitate the presence of probiotic LAB strains in the digestive tract of animals, including honeybees [74]. In our study, we examined the coaggregation of three LAB strains displaying the most potent adhesive properties (i.e., *L. plantarum* 18/1 and 21/1 and *P. acidilactici* 25/1) with seven honeybee pathogens (Table 2). Additionally, as a control strain, we used *A. kunkeei* DSM 12361. All the LAB strains were co-aggregated with pathogenic bacteria, but the degree of this phenomenon depended on the tested microorganism. *A kunkeei* DSM 12361 isolated from the honeybee gut showed the highest coaggregation with most of the pathogenic bacteria among the tested LAB, which proves the strong antagonism of this microorganism against honeybee pathogens. This strain showed a significantly more potent coaggregation ability with *P. larvae* ATCC 49843 in comparison to the *L. plantarum* strains (*p* < 0.05, KW test) (Table 2). A comparison of the results obtained for coaggregation to aggregation and hydrophobicity suggests no correlation between these properties. The known honeybee pathogens include the bacteria of the species *P. larvae*: the causing agents of American foulbrood disease [75]. The tested LAB strains showed a higher coaggregation with *P. larvae* ATCC 25367, ranging from 54.79% ± 4.36% for *L. plantarum* 18/1 to 89.37% ± 10.41% for *A. kunkeei* DSM 12361. *A kunkeei* DSM 12361 also demonstrated the highest coaggregation with *P. larvae* ATCC 49843, equal to 87.16% ± 1.27%. The obtained results suggest that the degree of coaggregation depends on the individual strain of the pathogen, regardless of whether it belongs to the same species. Subsequently, we investigated the coaggregation of LAB with *Paenibacillus apiarius* DSM 5582 and *Paenibacillus alvei* DSM 29, which negatively affects the viability of honeybee colonies by weakening the insects’ immune system through synergistic action with other pathogens [75,76]. The coaggregation of LAB with these Gram-positive, spore-forming, pathogenic bacteria ranged from 76.52% ± 1.17% for *L. plantarum* 18/1 to 89.61% ± 7.44% for *A. kunkeei* DSM 12361 and from 77.16% ± 4.24% for *L. plantarum* 18/1 to 85.74% ± 1.65% for *L. plantarum* 21/1, respectively. A pathogenic bacterium that also contributes to honeybee mortality is *Lysinibacillus sphaericus*, which causes population reduction in worker honeybees and lethal diseases in honeybee broods [77]. The highest coaggregation with *L. sphaericus* DSM 1866, equal to 97.13% ± 1.45%, was demonstrated by *L. plantarum* 18/1. Among the tested pathogens, LAB strains showed the weakest coaggregation with *Melissococcus plutonius* DSM 29964: the etiological agent of the European foulbrood of honeybees [78]. The coaggregation of LAB with this pathogen varied from 29.27% ± 8.74% for *A. kunkeei* to 44.82% ± 10.75% for *L. plantarum* 18/1. In the above study, *E. coli* ATCC 25922 was used as an opportunistic pathogen of honeybees and as a reference strain [79]. The highest coaggregation with this pathogen was demonstrated by *L. plantarum* 18/1 (96.06% ± 1.35%) and *A. kunkeei* DSM 12361 (93.09% ± 3.65%). The coaggregation capacity of LAB is time-dependent and strain-specific [74]. According to Li et al., the percentage of coaggregation of the five LAB strains with *E. coli*, *Salmonella enterica* subsp. *enterica* serovar Typhimurium and *Staphylococcus aureus* increased over time [74]. *Ligilactobacillus salivarius* M2-71 demonstrated the maximum percentage of coaggregation with *S.* Typhimurium and *E. coli* after 24 h of incubation [74]. Tatsaporn and Kornakon investigated the inhibition of *E. coli* ATCC 8739, *Bacillus cereus* ATCC 11778, and *Salmonella* Typhimurium ATCC 13311 biofilm formation through LABs isolated from chicken and fermented fish [10]. The highest percentage of coaggregation (74–80%) with all the pathogens tested was characteristic of *Enterococcus faecium* C6 [10]. Ekmekci et al. demonstrated that the percentage of LAB coaggregation with pathogens might depend on the incubation conditions [80]. Of the 19 LAB strains isolated from lateral vaginal walls, only *L. acidophilus* S1 showed potent coaggregation with *E. coli* ATCC 11229 under both anaerobic (62%) and aerobic conditions (71%) [80]. Due to the anaerobic conditions in the digestive tract of honeybees, the influence of environmental conditions on the growth and antimicrobial properties of potentially probiotic LAB should be further investigated through in vitro and in vivo tests [81]. According to the authors’ knowledge, the coaggregation of LAB with honeybee pathogens has not yet been thoroughly investigated.

### 2.4. Effect of LAB Cell-Free Supernatants (CFSs)

#### 2.4.1. On Adhesion of Honeybee Pathogens to Polystyrene

**Figure 5:**
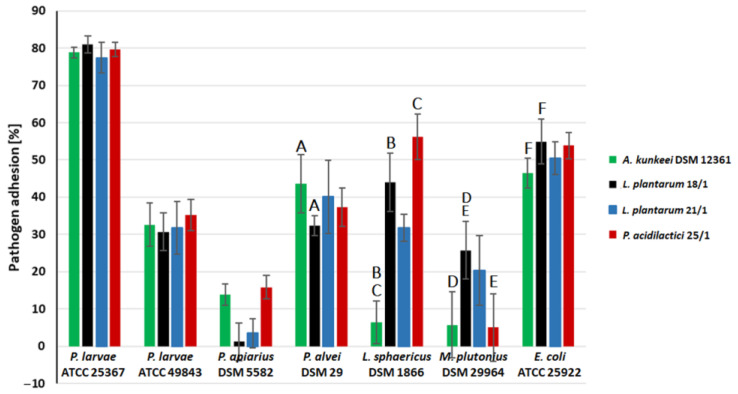
Effect of cell-free supernatants (CFSs) from lactic acid bacteria (LAB) on pathogen adhesion [%] to polystyrene after 1 h incubation. Each data point represents the mean from eight individual wells. The average absorbance of each pathogen incubated without CFSs was taken as 100%. Results are presented as mean ± standard deviation (SD). Statistically significant differences in the LAB effect on biofilm formation of honeybee pathogens are indicated on the chart with a letter, *p*-value as follows: A—*p* = 0.014; B—*p* = 0.004; C—*p* = 0.00004; D—*p* = 0.0053; E—*p* = 0.048; F—*p* = 0.0047.

In the next part of the study, we examined the effect of LAB CFSs (metabolites) on the adhesion capacity of seven honeybee pathogens (Figure 5) to a model polystyrene surface. CFSs from three LAB strains showed that the strongest adhesive properties were selected for this experiment (i.e., *L. plantarum* 18/1 and 21/1 and *P. acidilactici* 25/1). Additionally, *A. kunkeei* DSM 12361 was used as a control strain due to its natural occurrence in the digestive tract of honeybees. The anti-adhesive properties of CFSs were a strain-dependent feature and varied depending on the pathogen tested (Figure 5).

The most potent anti-adhesive properties of CFSs were observed for *P. apiarius* DSM 5582 and *M. plutonius* DSM 29964. The adhesion inhibition of these pathogens in the presence of LAB metabolites ranged from 84.17% ± 3.22% for *P. acidilactici* 25/1 to 98.80% ± 5.02% for *L. plantarum* 18/1 and from 74.23% ± 7.76 for *L. plantarum* 18/1 to 94.28% ± 8.82% for *P. acidilactici* 25/1, respectively. *A. kunkeei* DSM 12361 and *P. acidilactici* 25/1 significantly inhibited the biofilm formation of *M. plutonius* DSM 29964 adhesion in comparison to 18/1 (*p* < 0.05, KW test). The highest resistance to the anti-adhesion activity of CFSs was demonstrated by *P. larvae* ATCC 25367. The adhesion inhibition of this pathogen in the presence of CFSs ranged from 19.03% ± 2.28% for *L. plantarum* 18/1 to 22.56% ± 4.14% for *L. plantarum* 21/1. The tested CFSs more potently inhibited the adhesion of *P. larvae* ATCC 49843, suggesting that the level of LAB anti-adhesive properties varies against pathogen strains belonging to the same species. The strongest effect on the adhesion capacity of *P. larvae* ATCC 49843 was shown by CFSs from *L. plantarum* 18/1, where the inhibition of pathogen adhesion was equal to 30.70% ± 5.11%.

The tested CFSs also showed potent anti-adhesion activity against *P. alvei* DSM 29 and *L. sphaericus* DSM 1866. The adhesion of these pathogens was significantly lower in the presence of CFSs from *L. plantarum* 18/1 (32.38% ± 2.68%) and *A. kunkeei* DSM 12361 (6.48% ± 5.72%), respectively. The anti-adhesive properties against *L. sphaericus* DSM 1866 were statistically significant for *A. kunkeei* DSM 12361 in comparison to *L. plantarum* 18/1 and *P. acidilactici* 25/1 (*p* < 0.05, KW test). CFSs demonstrated moderate anti-adhesion activity against *E. coli* ATCC 25922. The adhesion of this pathogen in the presence of CFSs ranged from 46.51% ± 4.01% for *A. kunkeei* DSM 12361 to 54.91% ± 5.96% for *L. plantarum* 18/1. In the neutralized pH of CFSs, we did not observe any anti-adhesion activity (data not shown), indicating that the acidic pH provokes the inhibition of pathogen adhesion. This anti-adhesive activity may contribute to the inhibition of bacterial biofilm formation. CFSs are likely to inhibit the production of pathogen biofilms and protect the host organism from infection. Various LAB metabolites exhibit antagonistic activity against pathogens by acidifying the environment, and the pH mainly influences the adhesive properties of the bacteria [82,83].

El Hage et al. investigated the anti-adhesion activity of *L. reuteri* 1/c24 and *L. salivarius* A30/i26 and 16/c6 against three *Salmonella* serotypes (*S*. Infantis, *S*. Enteritidis, and *S*. Kentucky ST198) [84]. The competition assays showed that LAB displayed no effect on the adhesion of pathogens to Caco-2 cells [84]. In contrast, in vitro tests conducted by Jayashree et al. showed that *L. fermentum* MTCC significantly reduced the adhesion of *S. aureus* to Caco-2 cells, which suggests that the anti-adhesive properties of LAB are a strain-dependent feature [85]. Bulgasem et al. investigated the anti-adhesive properties of CFSs from 25 LAB strains isolated from honey against five *Candida* spp. [86]. In addition, the inhibition of pathogen adhesion by LAB metabolites depended on the pH of the supernatants and was stable after heat treatment [86]. According to Gutiérrez et al., the anti-adhesive activity of five fermented LAB broths showed strain-specific properties against seven potentially pathogenic microorganisms [87]. LAB metabolites regulated biofilm synthesis and reduced the adhesion of *B. cereus*, *E. coli* K92, and *Listeria innocua* [87].

#### 2.4.2. On Biofilm Eradication of Honeybee Pathogens

**Figure 6:**
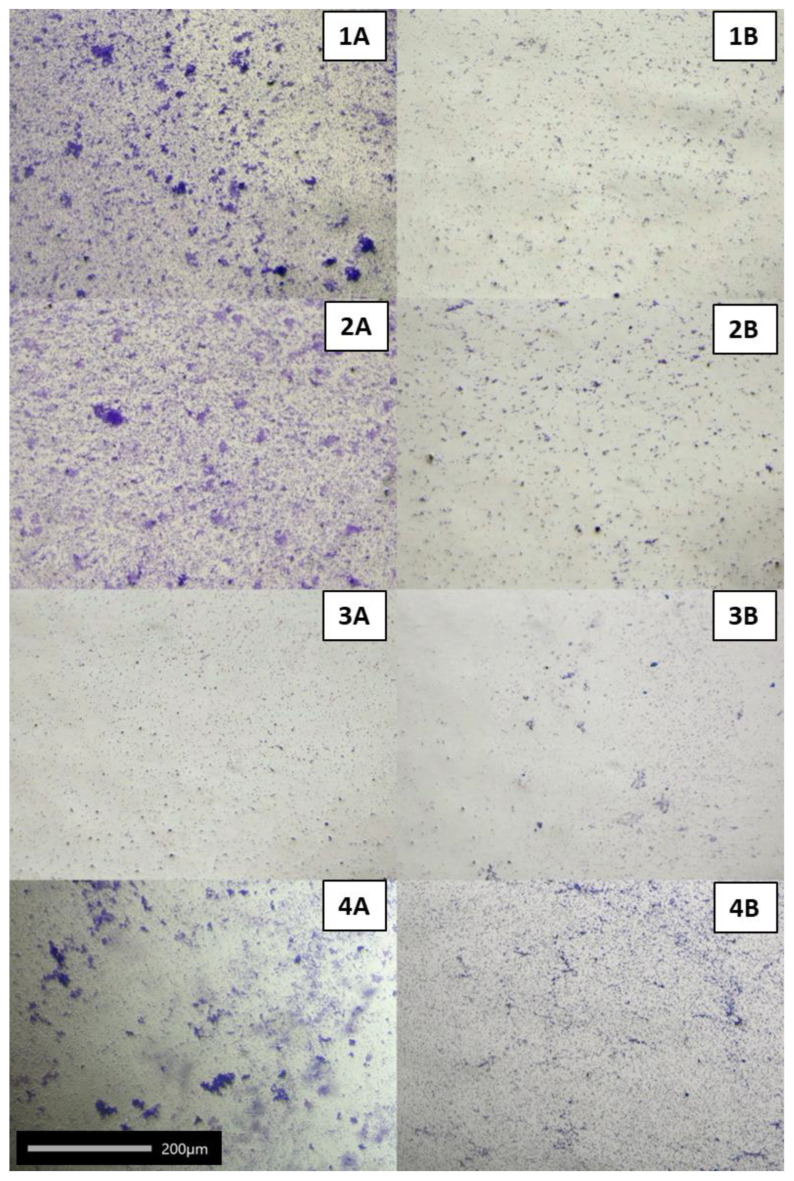
Selected microphotographs showing the effect of cell-free supernatants (CFSs) from *L. plantarum* 21/1 on biofilm eradication of: **1A** *P. larvae* ATCC 25367; **2A** *P. larvae* ATCC 49843; **3A** *L. sphaericus* DSM 1866; **4A** *M. plutonius* DSM 29964. **1**–**4B**: pathogen cells without CFSs (negative controls) (objective 20×). Observed under a phase-contrast microscope (Nikon Eclipse Ci H600L, Tokyo, Japan) attached to a digital camera (NikonDigital Sight DS-U3, Tokyo, Japan) and imaging software (NIS-elements BR 3.0, Nikon, Tokyo, Japan).

After confirming the anti-adhesive activity of the metabolites of *A. kunkeei* DSM 12361, *L. plantarum* 18/1 and 21/1, and *P. acidilactici* 25/1 against seven pathogenic bacteria, in this stage of the research, we checked the effect of CFSs (with physiological pH) on the mature pathogen biofilms (Table 3, Figure 6). The tested CFSs showed a weak eradication of pathogen biofilms. The strongest eradication of biofilms was observed for *M. plutonius* DSM 29964 and reached 19.87% ± 1.00% for CFSs from *L. plantarum* 18/1. *L. plantarum* 18/1 metabolites had the most potent effect on pathogen adhesion and demonstrated the ability to eradicate the biofilms of five out of seven microorganisms with statistical significance for *P. larvae* ATCC 49843, *M. plutonius* DSM 29964 and *E. coli* ATCC 25922. *L. plantarum* 21/1 also showed a strong biofilm eradication capacity of all the pathogens except for *P. larvae* ATCC 25367 and *P. alvei* DSM 29 (*p* < 0.05, KW test) (Table 3). For some pathogens, the incubation of the mature biofilm with CFSs resulted in additional growth stimulation, despite the acidic pH, as CFSs could probably provide a carbon source. Comparing the results of this experiment with the evaluation of the anti-adhesive capacity of CFSs, it could be suggested that LAB metabolites have a more limited effect on the existing biofilms than on the biofilm formation. Therefore, the prophylactic administration of probiotic strains with proven anti-adhesive properties to honeybees is highly justified in the prevention of infection. The eradication of microbial biofilms by conventional methods is often ineffective [88]. According to Davies [89], biofilm cells demonstrated resistance mechanisms against antimicrobial agents, which varied with the stage of the biofilm [89]. It is hypothesized that in pathogen biofilms, the formation of persister cells, nutrient limitation, and adaptive stress responses constitute a multi-layered defense [90]. All these factors may influence the resistance of biofilms to the eradication of LAB metabolites. According to the authors’ knowledge, the influence of CFSs on the formation and eradication of the biofilms of honeybee pathogens has not yet been thoroughly investigated. The anti-adhesive ability of LAB is a significant mechanism of colonialization resistance that defines these bacteria as probiotic microorganisms [91]. Some CFSs stimulated the formation of pathogenic biofilms (negative results in Table 3). This shows that pathogens in the biofilm can use LAB metabolites as a carbon source and that biofilm is even more abundant.

## 3. Materials and Methods

### 3.1. Chemicals, Vessels, and Other Materials

Tryptic Soy Broth (TSB), deMan, Rogosa, and Sharpe (MRS) Broth and agar, fructose, cysteine-hydrochloride, sodium chloride (NaCl), sodium hydroxide (NaOH), phosphate-buffered saline (PBS), high-glucose Dulbecco’s Modified Eagle Medium (DMEM), trypsin, 4-(2-hydroxyethyl)-1-piperazineethanesulphonic acid (HEPES), methanol, streptomycin–penicillin mixture for cell cultures, trypan blue, mucin from the porcine stomach, acetic acid, ethanol, gelatin, crystal violet, Triton X-100, and *n*-hexadecane were purchased from Merck Life Science, Warsaw, Poland. Anaerobe Basal Broth (ABB), foetal bovine serum (FBS), GlutaMAX^TM^, TrypLE^TM^ Express, and AnaeroGen^TM^ Atmosphere Generation Systems sachets were purchased from Thermo Fisher Scientific, Waltham, MA, USA. Cryobanks™ were from Copan Diagnostics Inc., Jefferson Avenue Murrieta, Murrieta, CA, USA. In addition, 6-, 24- and 96-well transparent flat-bottom plates, serological pipettes, and T75 roux flasks (all from Greiner Bio-One GmbH Kremsmünster, Austria) were purchased in Biokom Systems, Janki, Poland. Collagen-coated 96-well plates (BioCoat^®®^) were from Becton, Dickinson and Co., Franklin Lakes, NJ, USA. Disposable syringe filters (0.22 µm pore size) were purchased from Labindex S.A., Warsaw, Poland. The Caco-2 cell line was from Cell Line Service GmbH, Eppelheim, Germany.

### 3.2. Bacterial Strains and Growth Conditions

A total of 20 strains of LAB were used for this research. They were selected on the basis of the previous study; i.e., those with the strongest antagonistic activity towards honeybee pathogens, such as *P. larvae* or *M. plutonius* [83], and those with the highest efficiency of detoxification of insecticides were selected [92]. These were the following strains: *L. plantarum* (8AN, 145, 10/2, 18/1, 21/1), *P. acidilactici* (4/1, 5/2, 7/1, 8/1, 25/1), *P. pentosaceus* (11/3, 14/1, 19/1), *L. brevis* KKA, *L. casei* 12AN, *L. salivarius* 9AN, and *P. parvulus* OK-S. These were isolates from honeybee environments such as flowers, honey, or bee pollen (their isolation and characteristics were published previously [93], as well as the collection strains of different origins (e.g., fermented cucumbers) which were acquired from the collection of the Department of Environmental Biotechnology, Lodz University of Technology. *A. kunkeei* DSM 12361 was used as a reference strain, which is a symbiont naturally inhabiting the honeybee gut known to form abundant biofilms [42], was purchased from the German Collection of Microorganisms and Cell Cultures GmbH. The commercial probiotic strain *L. rhamnosus* GG was also used in the study as a positive control as it is known for its strong adhesive ability [94]. Furthermore, a negative control strain *L. rhamnosus* ŁOCK 0908, acquired from the Pure Culture Collection of the Institute of Fermentation Technology and Microbiology (ŁOCK 105), Lodz University of Technology, was also applied [61].

The following honeybee pathogens were used in the study: *P. larvae* ATCC 25367, *P. larvae* ATCC 49843, *E. coli* ATCC 25922 purchased from American Type Culture Collection; *P. alvei* DSM 29, *P. apiarius* DSM 5582, *L. sphaericus* DSM 1866, and *M. plutonius* DSM 29964 purchased from German Collection of Microorganisms and Cell Cultures GmbH (labeled as DSM).

All bacteria were stored in Cryobanks™ at −20 °C. Before the experiments, LAB strains were activated, threefold passaged (3% inoculum), and anaerobically cultured (with AnaeroGen Atmosphere Generation Systems sachets) in MRS broth for 24 h at 37 °C. *A. kunkeei* DSM 12361 was cultured anaerobically on MRS broth with the addition of fructose (10 g/L) and 0.05% cysteine-hydrochloride (MRS-F). *M. plutonius* DSM 29964 was cultured anaerobically in ABB medium, while the remaining pathogens were in TSB for 24 h at 37 °C. The active stock cultures were stored at 4 °C.

### 3.3. Preparation of Bacterial Suspensions

Prior to the experiment, overnight bacterial cultures were centrifuged (3852× *g*, 15 min, 4 °C), decanted, and suspended in PBS (pH 7.2). This operation was repeated until the culture medium was completely removed. Next, the final optical density of each suspension was established spectrophotometrically at 600 nm (Beckman DU 640 spectrophotometer, USA) to approximately 1.0 ± 0.1 by dilution in PBS.

### 3.4. Hydrophobicity and Auto-Aggregation of LAB

LAB suspensions in PBS (in three repeats each) in glass tubes were vortexed vigorously for 2 min with n-hexadecane (1:5, *v*/*v*) in order to form the emulsion. The mixtures were incubated for 60 min at ambient temperature until the hexadecane formed a separated layer above the PBS. The absorbance of the bottom layer (PBS) was measured at 600 nm. The percentage of hydrophobicity was calculated as follows:

$$\mathrm{Hydrophobicity}\;\left[\%\right]=\frac{A1-A2}{A1}\times 100$$

where A1 and A2 are the mean absorbance values before and after the extraction with hexadecane, respectively.

In the auto-aggregation test, LAB suspensions (in three repeats each) were put in a stable, safe place at an ambient temperature. After 24 h, the upper layer was collected for the measurement of the optical density. The percentage of aggregation was calculated as follows:

$$\mathrm{Auto}-\mathrm{aggregation}\;\left[\%\right]=\left[1-\left(\frac{A2}{A1}\right)\right]\times 100$$

where A1 is the initial mean absorbance of the suspension and A2 is the mean absorbance of the suspension after 24 h.

### 3.5. Adhesive Abilities

#### 3.5.1. To Abiotic Surfaces (PS and Glass)

LAB suspensions (in eight repeats each) in PBS were added into a 96-well polystyrene plate or a 6-well plate (in three repeats each) with a cover glass on the bottom of the well. They were incubated for 2 h at 37 °C, rinsed with water, fixed with 80% methanol (15 min), and stained with 0.1% crystal violet (15 min). Next, the wells were rinsed with water and incubated for 15 min with 30% acetic acid and shaking (120 r.p.m.) (LAUDA Varioshake VS 8 OE Shaker, Dr. R. Wobser Gmbh & Co., Lauda-Königshofen, Denmark). The absorbance was measured at 600 nm with a microplate reader (TriStar2 LB 942, Berthold Technologies GmbH and Co. KG, Bad Wildbad, Germany). To calculate the adhesion ratio of the bacteria to polystyrene or glass, the value of absorbance for the LAB strain was divided by the absorbance of the control sample (polystyrene or glass only). The adhesion capacity of a given strain was determined according to the following classification: A ≤ 1 no ability to adhesion; 2 ≥ A > 1 weak adhesion capacity; 3 ≥ A> 2 medium adhesion capacity; A > 3 strong adhesion ability.

#### 3.5.2. To Biotic Surfaces (Collagen, Mucus, and Gelatin)

The adhesion of bacteria to gelatin and mucous was determined by crystal violet staining. A 24-well plate was coated (60 min, 37 °C) with 1% sterile-filtered gelatin and left at 4 °C overnight. In the case of mucous, a 96-well plate was coated with mucous from the porcine stomach (72 h, 4 °C). The unattached gelatin or mucous were removed and rinsed with PBS. The bound mucous was fixed for 20 min at 60 °C. Next, bacterial suspensions in PBS were added to each well (in eight repeats each) and allowed to adhere for 2 h at 37 °C. Non-adherent bacteria were removed, washed with PBS, and the remaining were fixed (20 min, 60 °C). Then, the adhered bacteria were stained with 0.1% crystal violet (15 min), washed with PBS, and finally, a citrate buffer (20 mM/L; pH 4.3) was added to each well for 45 min with shaking (120 r.p.m.). The absorbance was determined at 600 nm. To calculate the adhesion ratio of the bacteria to gelatin/mucous, the value of bacterial absorbance was divided by the absorbance of the control sample (gelatin/mucous only). The methodology for adhesion to collagen was conducted as in the case of polystyrene with the usage of ready-to-use collagen-coated 96-well plates. The adhesion capacity of a given strain was determined according to the classification described in Section 3.5.1.

#### 3.5.3. To Human Colon Adenocarcinoma Cell Line Caco-2

The Caco-2 cells from the 44th passage were cultured in T75 Roux bottles in DMEM supplemented with 10% FBS, 25 mM HEPES, 4 mM Gluta^MAX^, 100 µg/mL streptomycin, and 100 IU/mL penicillin. The cells were incubated for 7 days at 37 °C in a 5% CO_2_ atmosphere and humidity >95% (Galaxy 48S, New Brunswick, United Kingdom). Then, they were detached from the substrate with Tryple^TM^ Express according to the manufacturer’s instructions. The cell suspension was centrifuged (209× *g*, 5 min), decanted, and the pellet was suspended in fresh DMEM. Then, the cell number was determined in a hemacytometer, and cell viability was determined by staining with trypan blue.

For the adhesion assay, Caco-2 cells were seeded into a 24-well plate at a concentration of 4.0 × 10^5^ cells/well and were left overnight. The next day, the overnight cultures of LAB strains were centrifuged (9300× *g*, 10 min), washed with sterile PBS, and centrifuged again until the culture medium was completely removed. The initial number of LAB added to the Caco-2 cell monolayer was evaluated for each strain by Koch’s plate method. Next, the bacterial pellets were mixed with DMEM without antibiotics. Simultaneously, DMEM was aspirated from Caco-2 cells in a 24-well plate, and 1 mL of the prepared suspensions of LAB in DMEM were added in three replications for each strain. The plate was incubated for 2 h at 37 °C in 5% CO_2_ and humidity >95%. After, the non-adhered LAB was removed by washing with PBS, whereas the adhered LAB together with Caco-2 cells were detached by adding 1% trypsin to each well and incubation (10 min, 37 °C). In the next step, adhered LAB with Caco-2 cells were scraped with a sterile cell scraper, transferred into sterile Eppendorf tubes, centrifuged (9300*×* g, 10 min), and, in order to lyse the Caco-2 cells, 0.1% Triton X-100 was added (5 min, room temperature). Adhering bacteria were evaluated with Koch’s plate method by counting colonies growing on the MRS agar after 48 h incubation at 37 °C. The adhesion rate was calculated as follows:

A [%]=(log A2/Log A1)×100

where A1 means the log CFU (colony forming units) of the initial LAB added to the well; A2 is the log CFU of adhered LAB.

For the microscopic visualization, after 2 h incubation, non-adherent bacteria were removed and washed with PBS, while adhered LAB was fixed with 70% methanol (15 min), stained (0.1% crystal violet), washed with 70% ethanol, and dried overnight. The prepared samples were observed at 20× objective under an inverted microscope Nikon Ts2 with EMBOSS contrast, integrated with Jenoptic Subra Full HD Color digital camera.

### 3.6. Coaggregation of Selected LAB with Honeybee Pathogens

For the coaggregation assay, cell suspensions were prepared in the same way as auto-aggregation, i.e., an equal volume of the LAB strain and pathogen suspension was vortexed for 10 s and then incubated for 24 h at room temperature. Control samples were single bacterial suspensions. Absorbance was measured after 24 h, as described above. The percentage of coaggregation was calculated as follows:

$$\mathrm{Coaggregation}\;\left(\%\right)=\frac{\frac{\left(A1-A2\right)}{2}-\;A3}{A3}\;\times \;100$$

where A1 is the initial mean absorbance of the pathogen suspension, A2 is the initial mean absorbance of the LAB suspension, and A3 is the mean absorbance of the bacterial mixture after 24 h.

### 3.7. Anti-Adhesion Ability of LAB Cell-Free Supernatants (CFSs)

#### 3.7.1. Preparation of CFSs

Overnight cultures of LAB were centrifuged (10,733× *g*, 15 min), and in the first option, the pH of the supernatants was measured. In the second option, the pH was adjusted to 7.0 ± 0.1 (with 0.1 M NaOH). Next, all the supernatants were sterile filtered with disposable syringe filters (0.22 μm) and frozen at −20 °C in falcons until analysis.

#### 3.7.2. Anti-Adhesion Test

This test was conducted for a model polystyrene surface, and CFSs of 3 LAB strains were selected on the basis of adhesion results plus control *A. kunkeei* DSM 12361. The following LAB strains with the strongest adhesion capacity were selected: *L. plantarum* 18/1 and 21/1 and *P. acidilactici* 25/1. The method was used as described in Section 3.5.1. with the difference that the pathogens were placed in the CFSs of each LAB, and the negative controls were the pathogens suspended in PBS. The incubation was conducted for 1 h. The percentage of the pathogen adhesion was calculated as follows:

$$\mathrm{Pathogen}\;\mathrm{adhesion}\;\left(\%\right)=\frac{A}{A_{p}}\times 100$$

where A is an average absorbance of a pathogen incubated with CFS and A_p_ is the average absorbance of a pathogen (without CFS).

### 3.8. Eradication of Pathogens Biofilm by LAB Strains

These tests were also evaluated for *L. plantarum* 18/1 and 21/1, *P. acidilactici* 25/1, and control strain *A. kunkeei* DSM 12361. Polystyrene flat-bottomed 96-well plates were inoculated with the appropriate pathogen (10% inoculum) in eight repeats each and were incubated for 7 days at 37 °C so that the bacteria could form a homogeneous biofilm. After this time, the medium with planktonic and loosely bound cells was aspirated from each well and washed gently with PBS. Then, 200 µL of CFSs for each LAB strain were added and incubated for 24 h at 37 ^o^C. The negative control was the biofilm of the pathogen alone with PBS. Next, the samples were aspirated, the wells were washed gently with PBS, air-dried (15 min), fixed with 80% methanol (15 min), stained with 0.1% crystal violet (10 min), washed with water, air-dried (15 min), and images of the biofilms were taken under an inverted microscope. The crystal violet was then extracted with 30% acetic acid with shaking (110 r.p.m.) for 15 min. Absorbance was measured at λ = 492 nm in a microplate reader. The percentage of biofilm eradication was calculated as follows:

$$\mathrm{Biofilm}\;\mathrm{eradication}\;\left(\%\right)=\frac{A_{24}}{A_{c}}\times 100$$

where A_24_ was an average of eight replicates of absorbance values at time t = 24 h and A_c_ was an average of eight replicates of absorbance values for the negative control at time t = 24 h.

### 3.9. Statistical Analysis

The results in Table 1 (the auto-aggregation, surface hydrophobicity of LAB strains), Table 2 (coaggregation of LAB strains with honeybee pathogens), and Table 3 (eradication “+” and stimulation “−“ of pathogen biofilms) are presented as the mean from three repeats ± standard error of the mean (S.E.M.). Table S1 in the Supplementary Materials (the comparison of LAB capacity to adhere to abiotic and biotic surfaces) presents data as the mean from eight repeats ± standard deviation (S.D.).

Non-parametric tests were used for statistical analyses of the analyzed parameters as auto-aggregation, surface hydrophobicity, eradication surface hydrophobicity, and adhesion to surfaces values of LAB did not follow a normal distribution (Shapiro–Wilk test). Differences regarding the analyzed parameters were tested using the Kruskal–Wallis test (KW test), followed by a multiple comparison test (MCT) to indicate significant differences between the groups. The correlation between the auto-aggregation and hydrophobicity of the tested LAB strains was analyzed using Spearman’s rank correlation coefficient.

A *p-*value < 0.05 was considered statistically significant. The KW test, UMW test, and Spearman’s rank correlation analyses were performed using Statistica ver. 13.1 (StatSoft, Tulsa, OK, USA).

## 4. Conclusions

In the above study, all the tested LAB strains showed varying levels of auto-aggregation and surface hydrophobicity related to pathogen displacement and adhesion. *L. plantarum* 18/1, *P. pentosaceus* 19/1, and *P. acidilactici* 25/1 showed the highest auto-aggregation and hydrophobicity of all the tested strains. No correlation was observed between the auto-aggregation capacity and the levels of hydrophobicity. Most of the LAB strains showed higher auto-aggregation and hydrophobicity than the probiotic *L. rhamnosus* GG and *A. kunkeei* DSM 12361. All LAB strains demonstrated the ability to adhere to the tested surfaces, with the highest values observed for glass. It was also observed that the level of adhesion differed between the strain and the surface, demonstrating the broad spectrum of LAB adhesion abilities. The origin of the strain did not affect its adhesion to Caco-2 cells, where *P. acidilactici* 5/2 showed the highest adhesion capacity. The strains showing the strongest adhesion abilities were *P. acidilactici* 25/1 and *L. plantarum* 18/1 and 21/1. The ability of the bacteria to co-aggregate with honeybee pathogens may contribute to strengthening the resistance of these insects. Co-aggregation was a strain-dependent feature and differed depending on the pathogenic bacteria tested. LAB strains showed the highest co-aggregation with *L. sphaericus* DSM 1866. The different results obtained for the co-aggregation of LAB with *P. larvae* ATCC 25367 and ATCC 49843 suggest that this is a unique feature in relation to pathogen strains belonging to the same species. All the tested CFSs strains showed a broad spectrum of anti-adhesive activity against the tested pathogens. The strongest inhibition of adhesion was observed for *P. apiarius* DSM 5582 and *M. plutonius* DSM 29964. The low biofilm eradication shown by CFSs suggests a weak effect of LAB metabolites on mature biofilms of pathogenic microorganisms.

The anti-adhesive activity demonstrated by LAB may contribute to the biocontrol of pathogens at an early stage of infection. LAB strains that show the highest adhesive capacity and the strongest anti-adhesive activity against the pathogens tested will be selected for future in vitro tests, such as antibiotic resistance and survival in sugar syrups or simulated gastrointestinal conditions. The above study requires confirmation in vivo and suggests the potential of LAB to enhance the resistance of honeybees to various pathogenic bacteria.

## Figures and Tables

**Table 1:** Auto-aggregation [%] and surface hydrophobicity [%] of LAB strains. The presented results are the mean from three repeats ± standard error of the mean (S.E.M.).

LAB Strains	Auto-Aggregation [%] ± S.E.M.	Hydrophobicity [%] ± S.E.M.
*A. kunkeei* DSM 12361	32.91 ± 9.06	18.87 ± 0.94
*P. acidilactici* 4/1	27.74 ± 4.50	1.83 ± 0.46 ^a,b^
*P. acidilactici* 5/2	52.58 ± 4.47	21.65 ± 3.66
*P. acidilactici* 7/1	52.09 ± 7.90	26.49 ± 5.12
*P. acidilactici* 8/1	33.74 ± 9.06	33.64 ± 6.09
*L. plantarum* 10/2	61.61 ± 3.12	45.13 ± 4.76
*P. pentosaceus* 11/3	52.03 ± 9.75	31.31 ± 1.99
*P. pentosaceus* 14/1	68.25 ± 5.97	19.85 ± 2.86
*L. plantarum* 18/1	71.91 ± 5.44	52.45 ± 2.80
*P. pentosaceus* 19/1	68.04 ± 5.31	63.16 ± 5.27 ^a^
*L. plantarum* 21/1	66.63 ± 3.00	47.05 ± 4.12
*P. acidilactici* 25/1	65.04 ± 0.90	73.49 ± 2.72 ^b^
*P. parvulus* OK-S	65.56 ± 2.71	37.43 ± 2.78
*L. brevis* KKA	54.68 ± 7.18	22.69 ± 4.69
*L. plantarum* 8AN	54.63 ± 3.53	22.45 ± 8.40
*L. salivarius* 9AN	53.98 ± 3.08	27.50 ± 2.81
*L. casei* 12AN	59.47 ± 4.56	14.31 ± 0.89
*L. plantarum* 145	30.96 ± 0.69	4.77 ± 2.01
*L. rhamnosus* GG	60.83 ± 0.75	16.43 ± 2.80
*L. rhamnosus* ŁOCK 0908	53.87 ± 0.52	13.03 ± 4.90

^a^—*p* = 0.044; ^b^—*p* = 0.015.

**Table 2:** Coaggregation [%] of LAB strains with honeybee pathogens after 24 h incubation. The presented results are the mean from three repeats ± standard error of the mean (S.E.M.).

Honeybee Pathogens	Coaggregation [%] ± S.E.M.				
	*A. kunkeei* DSM 12361	*L. plantarum* 18/1	*L. plantarum* 21/1	*P. acidilactici* 25/1	*p*-Value (KW Test)
*P. larvae* ATCC 25367	89.37 ± 10.41	54.79 ± 4.36	55.77 ± 7.51	80.40 ± 5.95	*p* = 0.075
*P. larvae* ATCC 49843	87.16 ± 1.27 *	42.96 ± 1.38 *	47.04 ± 2.29	48.33 ± 2.56	*p* = 0.043
*P. apiarius* DSM 5582	89.61 ± 7.44	76.52 ± 1.17	86.12 ± 5.49	80.53 ± 6.57	*p* = 0.264
*P. alvei* DSM 29	84.70 ± 3.69	77.16 ± 4.24	85.74 ± 1.65	77.22 ± 5.31	*p* = 0.459
*L. sphaericus* DSM 1866	96.11 ± 3.52	97.13 ± 1.45	95.15 ± 7.76	73.86 ± 1.90	*p* = 0.092
*M. plutonius* DSM 29964	29.27 ± 8.74	44.82 ± 10.75	43.22 ± 13.14	39.20 ± 2.90	*p* = 0.281
*E. coli* ATCC 25922	93.09 ± 3.65	96.06 ± 1.35	61.97 ± 0.51	60.06 ± 2.65	*p* = 0.072

* *p* = 0.028 (MCT) between *A. kunkeei* DSM 12361 and *L. plantarum* 18/1.

**Table 3:** Eradication (+) and stimulation (−) of pathogen biofilms [%] by cell-free supernatants (CFSs) from lactic acid bacteria (LAB) after 24 h incubation. Letters A, B and C indicate the pairs of CFS for which the eradication properties of pathogen biofilms were significantly different. The presented results are the mean from eight individual wells ± standard error of the mean (S.E.M.).

Honeybee Pathogens	Eradication (+) and Stimulation (−) of Pathogen Biofilms [%] ± S.E.M.				*p-*Value KW Test
	*A. kunkeei* DSM 12361	*L. plantarum* 18/1	*L. plantarum* 21/1	*P. acidilactici* 25/1	
*P. larvae* ATCC 25367	−4.86 ± 4.31	−8.91 ± 6.68	−18.18 ± 2.53	−15.50 ± 4.58	
*P. larvae* ATCC 49843	−5.43 ± 2.88 ^A^	10.36 ± 1.90 ^B^	17.53 ± 2.41 ^A,C^	−10.13 ± 4.12 ^B,C^	^A^ 0.0092, ^B^ 0.0451, ^C^ 0.0007
*P. apiarius* DSM 5582	−29.96 ± 2.79 ^A^	−5.04 ± 3.21	0.12 ± 3.94 ^A,B^	−27.27 ± 3.32 ^B^	^A^ 0.008, ^B^ 0.028
*P. alvei* DSM 29	−8.10 ± 3.10	−11.14 ± 5.50	−8.37 ± 4.50	−5.47 ± 6.88	
*L. sphaericus* DSM 1866	−0.35 ± 3.69 ^A^	0.99 ± 2.26	16.31 ± 2.51 ^A,B^	−6.73 ± 4.54 ^B^	^A^ 0.028, ^B^ 0.003
*M. plutonius* DSM 29964	−16.89 ± 6.28	19.87 ± 1.00 ^A^	19.33 ± 2.28 ^B^	−32.59 ± 4.31 ^A,B^	^A^ 0.003, ^B^ 0.004
*E. coli* ATCC 25922	−42.30 ± 6.76 ^A,B^	8.52 ± 2.74 ^A^	9.22 ± 2.96 ^B^	−7.04 ± 3.00	^A^ 0.003, ^B^ 0.001

## Data Availability

The data presented in this study are available in this article and from the corresponding authors upon reasonable request.

## Supplementary Materials

The following supporting information can be downloaded at: https://www.mdpi.com/article/10.3390/molecules27248945/s1, Table S1: The comparison of lactic acid bacteria (LAB) capacity to adhere to abiotic and biotic surfaces. The presented results are the mean from eight repeats ± standard deviation (S.D.). Statistically significant differences are indicated with “*” or “**” (see the legend). Additional information on the *p*-values in comparison with specific LAB strains is presented in the table below.

## Sample Availability

Samples of the compounds are not available from the authors.
