# Supplementary material for: Adhesion and Anti-Adhesion Abilities of Potentially Probiotic Lactic Acid Bacteria and Biofilm Eradication of Honeybee (Apis mellifera L.) Pathogens
Source: Molecules. 2022 Dec 15;27(24):8945. doi: 10.3390/molecules27248945 (PMC9786635; doi:10.3390/molecules27248945)
Supplement: Supplementary file 1 [file molecules-27-08945-s001.zip › molecules-2101148-supplementary.pdf]

Supplementary Materials

Adhesion and Anti-Adhesion Abilities of Potentially Probiotic Lactic Acid Bacteria and Biofilm Eradication of Honeybee (*Apis mellifera* L.) Pathogens

Aleksandra Leska [1,], Adriana Nowak [1,*] and Karolina Henryka Czarnecka-Chrebelska [2]

[1] Department of Environmental Biotechnology, Lodz University of Technology, Wolczanska 171/173, 90-530 Lodz, Poland
[2] Department of Biomedicine and Genetics, Medical University of Lodz, 5 Mazowiecka Str. (A-6 Building), 92-215 Lodz, Poland
* Correspondence: adriana.nowak@p.lodz.pl

Table S1. The comparison of lactic acid bacteria (LAB) capacity to adhere to abiotic and biotic surfaces. The presented results are mean from eight repeats ± standard deviation (S.D.). Statistically significant differences are indicated with "*" or "**" (see the legend). Additional information on the *p*-values in comparison between specific LAB strains is presented below the table.

LAB strains	Polystyrene	Glass	Collagen	Gelatin	Mucus	Caco-2 cells
A. kunkeei DSM 12361	1.12 ± 0.1	1.32 ± 0.07	0.96 ± 0.12 *	1.07 ± 0.07	1.24 ± 0.28 **	92.39 ± 1.55
P. acidilactici 4/1	1.01 ± 0.11	1.93 ± 0.22	1.08 ± 0.15	1.1 ± 0.17	1.18 ± 0.18 **	75.58 ± 2.45
P. acidilactici 5/2	1.48 ± 0.11	2.45 ± 0.82	1.11 ± 0.09	1.48 ± 0.11 **	1.02 ± 0.14	93.47 ± 0.37 **
P. acidilactici 7/1	1.54 ± 0.22	3.03 ± 0.15	1.17 ± 0.15	1.31 ± 0.14	1.14 ± 0.26	82.3 ± 2.48
P. acidilactici 8/1	1.23 ± 0.18	2.91 ± 0.34	1.15 ± 0.14	1.19 ± 0.2	0.85 ± 0.15	84.95 ± 3.36
L. plantarum 10/2	1.21 ± 0.16	1.84 ± 0.17	1.09 ± 0.13 *	1.25 ± 0.2	1.04 ± 0.34	56.75 ± 4.03*
P. pentosaceus 11/3	1.47 ± 0.17	3.3 ± 0.21 **	1.21 ± 0.17	1.23 ± 0.08	1.01 ± 0.28	54.88 ± 3.73*
P. pentosaceus 14/1	1.79 ± 0.4 **	2.88 ± 0.63	1.58 ± 0.16 **	1.69 ± 0.3 **	1.16 ± 0.23 **	91.36 ± 0.61
L. plantarum 18/1	2.38 ± 0.53 **	1.81 ± 0.04	1.37 ± 0.07 **	1.17 ± 0.13	1.13 ± 0.25	90.4 ± 1.1
P. pentosaceus 19/1	1.75 ± 0.1 **	0.99 ± 0.13	1.32 ± 0.13 **	1.08 ± 0.3	1.03 ± 0.16	87.41 ± 1.03
L. plantarum 21/1	1.95 ± 0.19 **	2.06 ± 0.24	1.55 ± 0.14 **	0.98 ± 0.07 *	1.06 ± 0.14	92.98 ± 0.94**
P. acidilactici 25/1	2.86 ± 0.22 **	2.32 ± 0.15	1.81 ± 0.19 **	1.3 ± 0.61	1.23 ± 0.2 **	83.38 ± 2.02
P. parvulus OK-S	0.95 ± 0.24 *	2.33 ± 0.39	1.02 ± 0.1 *	1.01 ± 0.11 *	0.95 ± 0.18	83.48 ± 4.03
L. brevis KKA	0.98 ± 0.07 *	1.97 ± 0.43	1.08 ± 0.12 *	0.95 ± 0.08 *	0.93 ± 0.14	77.3 ± 3.93
L. plantarum 8AN	1.31 ± 0.12	0.9 ± 0.05 *	0.96 ± 0.13 *	0.98 ± 0.08 *	0.77 ± 0.07	78.43 ± 0.89
L. salivarius 9AN	1.01 ± 0.17	1.94 ± 0.18	1.12 ± 0.16	1.12 ± 0.14	0.91 ± 0.07	77.95 ± 3.44
L. casei 12AN	1.63 ± 0.17 **	2.66 ± 1.04	1.22 ± 0.17	1.63 ± 0.17 **	1.04 ± 0.1	90.79 ± 0.76
L. plantarum 145	1.28 ± 0.15 *	2.12 ± 0.88	1.39 ± 0.14 **	1.18 ± 0.06	1.03 ± 0.1	75.84 ± 1.72
L. rhamnosus GG	1.4 ± 0.11	2.39 ± 0.14	1.2 ± 0.19	1 ± 0.06 *	1.18 ± 0.19 **	91.04 ± 0.69
L. rhamnosus ŁOCK 0908	1.31 ± 0.1	2.7 ± 0.36	1.1 ± 0.16	0.72 ± 0.08 *	1.39 ± 0.32 **	69.29 ± 0.61

* significantly lower adhesion capacity on the given surface (p <0.05. KW test)
** significantly increased adhesion capacity on the given surface (p <0.05. KW test)

For polystyrene: *L. salivarius* 9AN, *L. brevis* KKA, *P. parvulus* OK-S, *P. acidilactici* 4/1 were significantly decreased in comparison to the *L. plantarum* 18/1; *P. pentosaceus* 19/1; *L. plantarum* 21/1; *P. acidilactici* 25/1, *P. pentosaceus* 14/1; *L. casei* 12AN.

p-values for *L. salivarius* 9AN: 0,000168; 0,003199; 0,000332; 0,000005; 0,012401; 0,031482 (respectively);

p-values for *L. brevis* KKA: 0,000043; 0,000962; 0,000089; 0,000001; 0,004027; 0,010812 (respectively);
p-values for *P. parvulus* OK-S: 0,000036; 0,000813; 0,000074; 0,000001; 0,003441; 0,009310 (respectively);
p-values for *P. acidilactici* 4/1: 0,000105; 0,002106; 0,000209; 0,000003 (respectively).

For glass: *L. plantarum* 8AN was significantly decreased in comparison to the *P. pentosaceus* 11/3 (*p* value = 0.038, KW test).

For collagen: *L. plantarum* 8AN; *L. brevis* KKA; *P. parvulus* OK-S, *L. plantarum* 10/2, *A. kunkeei* DSM 12361 were significantly decreased in comparison to the *L. plantarum* 145; *L. plantarum* 18/1; *P. pentosaceus* 19/1; *L. plantarum* 21/1; *P. acidilactici* 25/1; *P. pentosaceus* 14/1.
p-values for *L. plantarum* 8AN: 0,003445; 0,002762; 0,039621; 0,000085; 0,000004, 0,000116 (respectively);
p-values for *L. brevis* KKA: 0,015014; 0,001300; 0,016391 (respectively);
p-values for *P. parvulus* OK-S: 0,017583; 0,014347; 0,000574; 0,000034; 0,000715 (respectively);
p-values for *L. plantarum* 10/2: 0,026521; 0,002471; 0,028333 (respectively);
p-values for *A. kunkeei* DSM 12361: 0,003011; 0,002410; 0,035199; 0,000073; 0,000003 (respectively).

For gelatin: *L. plantarum* 8AN; *L. brevis* KKA; *P. parvulus* OK-S; *L. plantarum* 21/1; *L. rhamnosus* GG; *L. rhamnosus* ŁOCK 0908 were significantly decreased in comparison to the *P. acidilactici* 5/2, *P. pentosaceus* 14/1, *L. casei* 12A.
p-values for *L. plantarum* 8AN: 0,004036; 0,002845; 0,000828 (respectively);
p-values for *L. brevis* KKA: 0,001854; 0,001326; 0,000360 (respectively);
p-values for *P. parvulus* OK-S: 0,036750; 0,025062; 0,008951 (respectively);
p-values for *L. plantarum* 21/1: 0,007249; 0,005059; 0,001554 (respectively);
p-values for *L. rhamnosus* GG: 0,015709; 0,010833; 0,003574 (respectively);
p-values for *L. rhamnosus* 0908: 0,000002; 0,000002; 0,000000 (respectively).

For mucus: *L. plantarum* 8AN was significantly decreased in comparison to the *P. acidilactici* 4/1; *P. acidilactici* 25/1; *A. kunkeei* DSM 12361; *L. rhamnosus* GG; *L. rhamnosus* 0908; *P. pentosaceus* 14/1.

p-values for *L. plantarum* 8AN: 0,009418; 0,002383; 0,004997; 0,013126; 0,000294; 0,042484 (respectively).

For Caco-2 cells: *L. plantarum* 10/2. *P. pentosaceus* 11/3 adhesion capacities were significantly decreased in comparison to the *P. acidilactici* 5/2.
p-value for *L. plantarum* 10/2: 0.032027;
p-value for *P. pentosaceus* 11/3: 0.024098.
P. pentosaceus 11/3 adhesion capacity was significantly decreased in comparison to the *L. plantarum* 21/1.
p-value for *P. pentosaceus* 11/3: 0.046460.
